# Supplementary material for: Fabrication, DFT Calculation, and Molecular Docking of Two Fe(III) Imine Chelates as Anti-COVID-19 and Pharmaceutical Drug Candidate
Source: Int J Mol Sci. 2022 Apr 3;23(7):3994. doi: 10.3390/ijms23073994 (PMC8999679; doi:10.3390/ijms23073994)
Supplement: Supplementary file 1 [file ijms-23-03994-s001.zip › ijms-1660002-supplementary.pdf]

Fabrication, DFT calculation and Molecular docking of two Fe(III) imine chelates as Anti-COVID-19 and pharmaceutical Drug Candidate

Hany M. Abd El-Lateef [1,2], Mai M. Khalaf[1,2], Mohamed R. Shehata[3], Ahmed M. Abu-Dief[1,4]*

[1]Department of Chemistry, College of Science, King Faisal University, P.O. Box 400 Al-Ahsa 31982, Saudi Arabia

[2]*Chemistry Department, Faculty of Science, Sohag University, Sohag-82534, Egypt.*

[4]*Chemistry Department, Faculty of Science, Cairo University, Giza, Egypt*

[4]*Chemistry Department, College of Science, Taibah University, Madinah, P.O. Box 344, Saudi Arabia.*

*** Corresponding authors: amamohammed@taibahu.edu.sa; ahmed_benzoic@yahoo.com (A. M. Abu-Dief) hmahmed@kfu.edu.sa (H. M. Abd El-Lateef);**

Supporting Information:

Table S1. Molecular electronic spectra, λ_{max} (nm) and ε_{max} (dm^3 mol^{-1} cm^{-1}) of the prepared PDBS, CPBS imine Ligands and their Fe^{3+} chelates in EtOH at 298 K against EtOH as a blank.

Compounds	λ_{max}(nm)	$\varepsilon_{max,}$ (dm^3 Mol^{-1} cm)	Assignment
**PDBS**	**254**<br>**292**<br>**384**	**1875**<br>**1750**<br>**1851**	**Π- Π***<br>**n- Π***<br>**intra ligand**
**PDBSFe**	**238**<br>**398**<br>**478**<br>**530**	**2587**<br>**2398**<br>**2922**<br>**1219**	**Π- Π***<br>**n- Π***<br>**MLCT**<br>**d-d**
**CPBS**	**235**<br>**386**<br>**440**	**2547**<br>**2378**<br>**2503**	**Π- Π***<br>**n- Π***<br>**Intraligand**
**CPBSFe**	**238**<br>**394**<br>**464**<br>**512**	**2573**<br>**2325**<br>**2080**<br>**1116**	**Π- Π***<br>**n- Π***<br>**MLCT**<br>**d-d**

Table S2. The antimicrobial results for PDBS, CPBS imine Ligands and their Fe^{3+} chelates.

**Compounds**	**Inhibition zone (mm)**						**± SD**					
	Serratia Marcescence (-ve)		*Escherichia Coli (-ve)*		*Micrococcus Luteus (+ve)*		*Aspergillus Flavus*		*Getrichm Candidum*		*Fusarium Oxysporum*	
Conc. (μg/ml)	**10**	**20**	**10**	**20**	**10**	**20**	**10**	**20**	**10**	**20**	**10**	**20**
**PDBS**	5.3±0.11	8.8±0.07	4.55±0.12	6.9±0.05	7.35±0.08	10.85±0.11	4.22±0.07	7.9±0.14	8.4±0.10	13.5±0.21	6.35±0.16	9.25±0.13
**PDBSFe**	12.4 ±0.15	26.7±0.10	10.2 ±0.05	20.5±0.13	17.55±0.10	37.85±0.23	8.9±0.03	16.7±0.31	14.85±0.21	35.3±0.13	11.8±0.17	23.9±0.12
**CPBS**	7.30±0.08	10.5±0.32	6.2±0.10	9.10±0.08	9.6±0.11	12.75±0.10	6.5±0.11	10.25±0.21	10.3±0.23	15.4±0.15	8.4±0.10	11.50±0.02
**CPBSFe**	13.80±0.21	29.4±0.13	11.1±0.17	23.6±0.15	18.90±0.11	39.7±0.10	10.35±0.12	18.6±0.21	16.9±0.25	35.8±0.12	12.4±0.15	25.7±0.13
**Ofloxacin**	16.50±0.11	33.20±0.20	15.5±0.05	27.6±0.15	24.80±0.13	45.70±0.11						
**Fluconazol**							14.70±0.20	24.3.±0.09	23.6±0.06	41.4±0.07	16.60±0.11	29.3±0.25

Table S3. Antimicrobial activity index (percentage) of the designated DPBS and CPBS ligands and their metal chelates

Compounds	Activity index (%)					
	Bacteria			Fungi		
	S. marcescence	*E. coli*	*M .luteus*	*A. flavus*	*G. candidum*	*F. oxysporum*
**PDBS**	**26.51**	**25.00**	**23.74**	**32.51**	**32.85**	**31.57**
**PDBSFe**	**80.42**	**74.28**	**82.82**	**68.72**	**85.89**	**78.50**
**CPBS**	**31.63**	**32.97**	**27.79**	**42.18**	**37.47**	**39.25**
**CPBSFe**	**88.55**	**85.51**	**86.88**	**76.54**	**87.10**	**87.71**

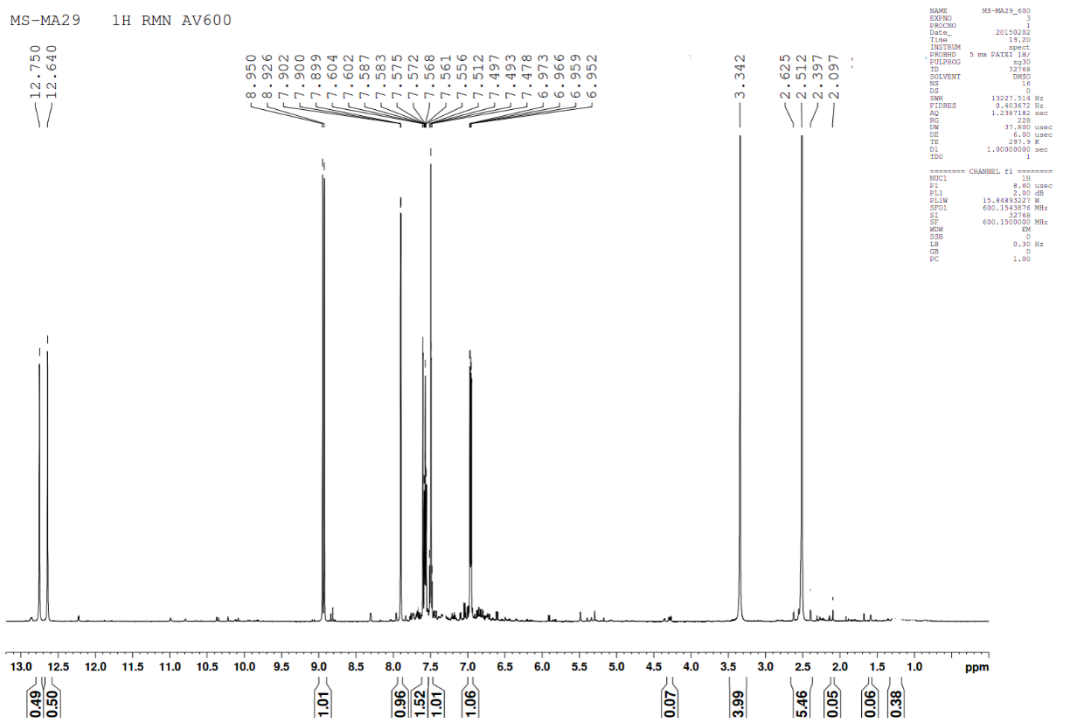

Figure S1. ¹H NMR spectrum of CPBS ligand

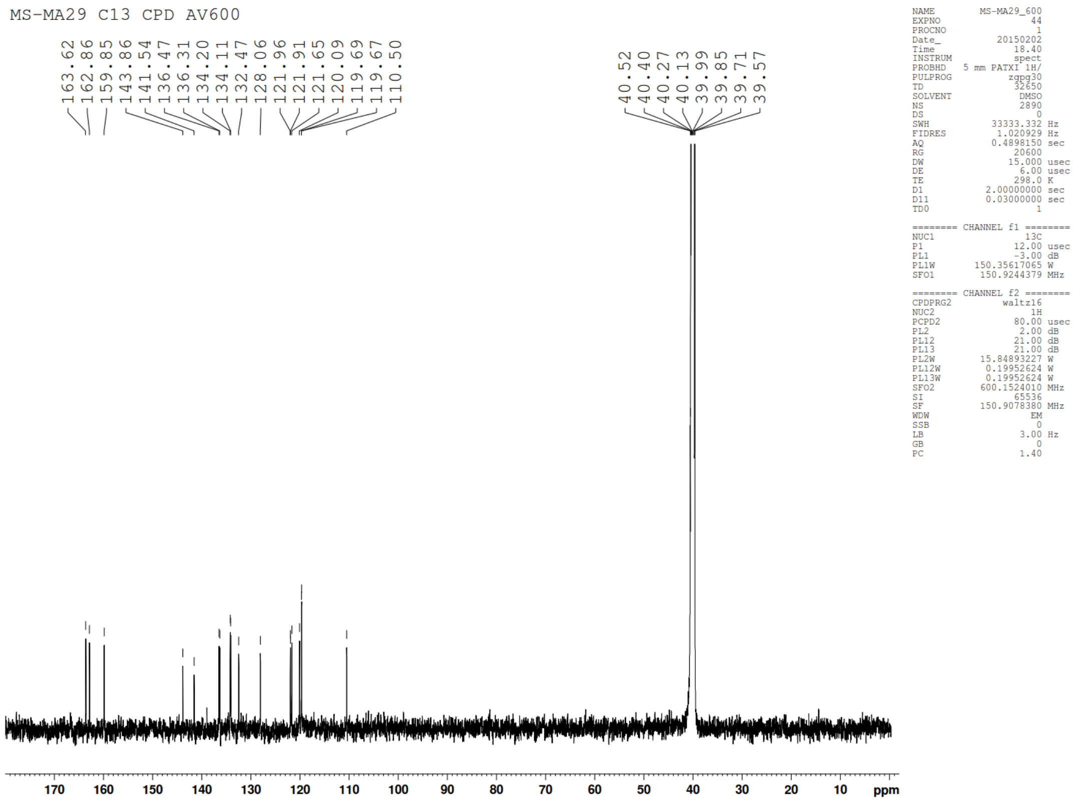

Figure S2. ¹³C NMR spectrum of CPBS ligand

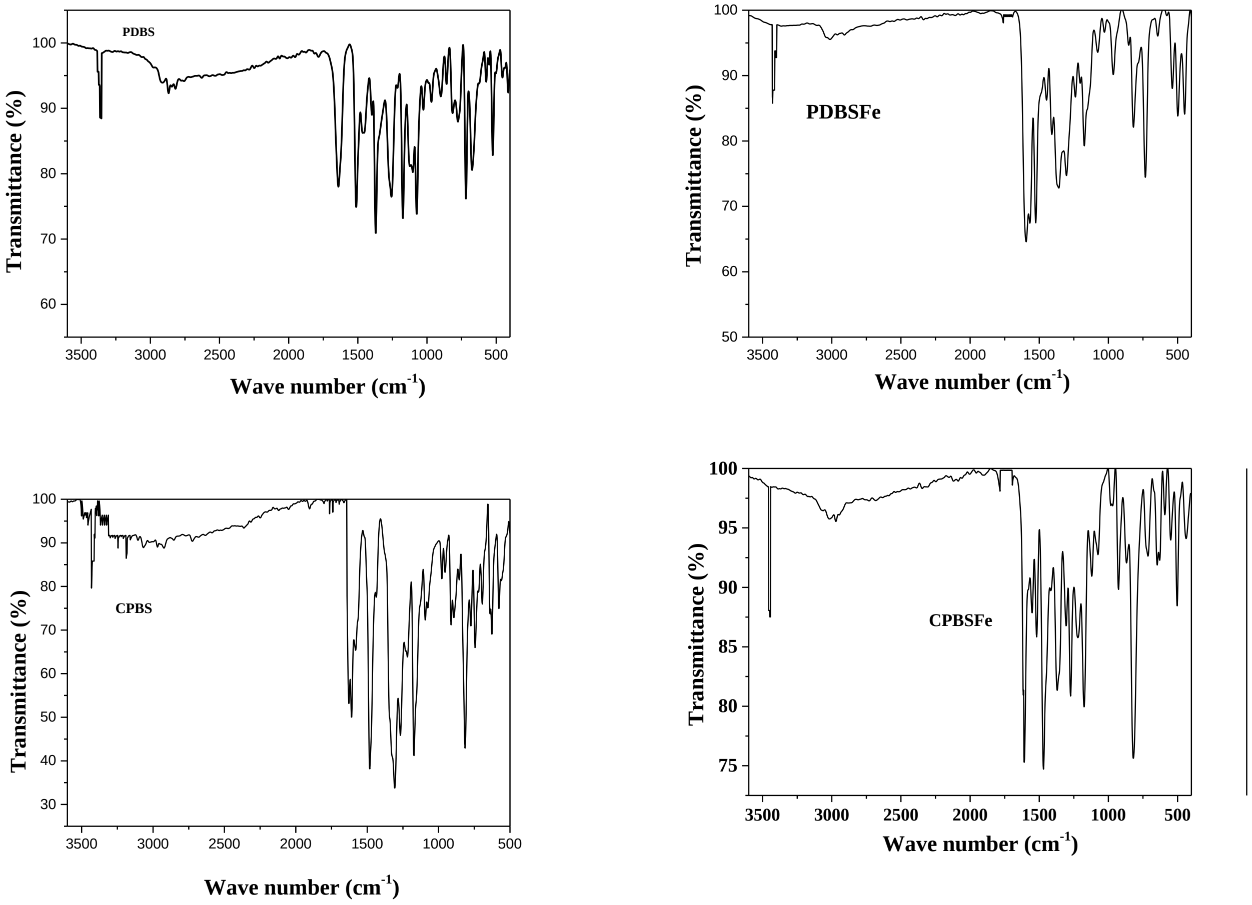

Figure S3. IR spectroscopy of PDBS, CPBS ligands and their Fe(III) chelates.

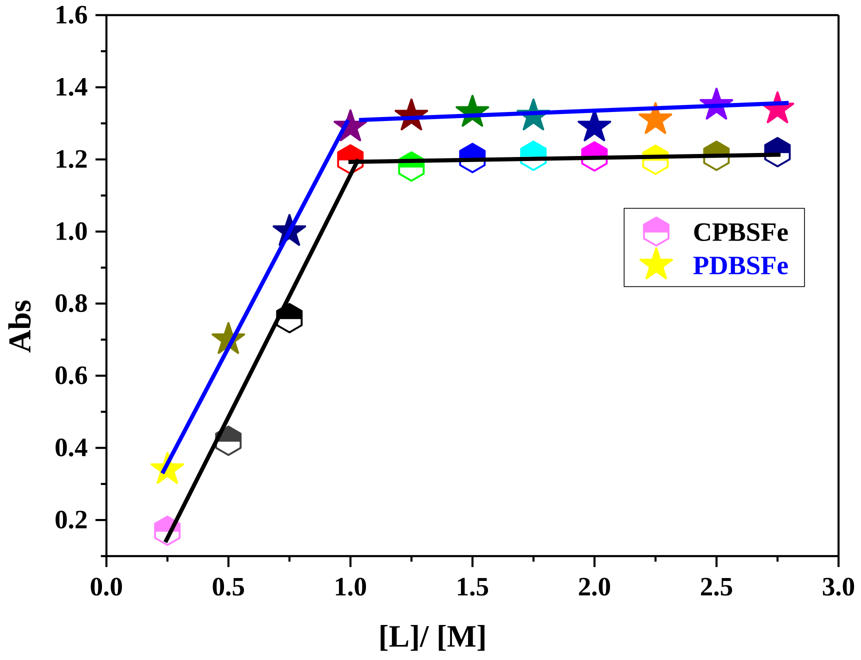

Figure S4. Molar ratio plots for the studied complexes in aqueous–ethanolic mixture at [Fe^{3+}] = 10^{-3}M and [L] = 10^{-3} M.
